# Supplementary material for: Development of folate receptor targeting chimeras for cancer selective degradation of extracellular proteins
Source: Nat Commun. 2024 Oct 8;15:8695. doi: 10.1038/s41467-024-52685-9 (PMC11461649; doi:10.1038/s41467-024-52685-9)
Supplement: Supplementary file 1 — Supplementary Information [file 41467_2024_52685_MOESM1_ESM.pdf]

# Supplementary information

## Development of folate receptor targeting chimeras for cancer selective degradation of extracellular proteins

Yaxian Zhou[1], Chunrong Li[1], Xuankun Chen[1], Yuan Zhao[1], Yaxian Liao[2], Penghsuan Huang[2], Wenxin Wu[2], Nicholas S. Nieto[1], Lingjun Li[1,2] and Weiping Tang[1,2*]

[1]Lachman Institute of Pharmaceutical Development, School of Pharmacy, University of Wisconsin-Madison, Madison, WI, USA 53705.

[2]Department of Chemistry, University of Wisconsin-Madison, Madison, WI, USA 53706.

[*]Correspondence: weiping.tang@wisc.edu

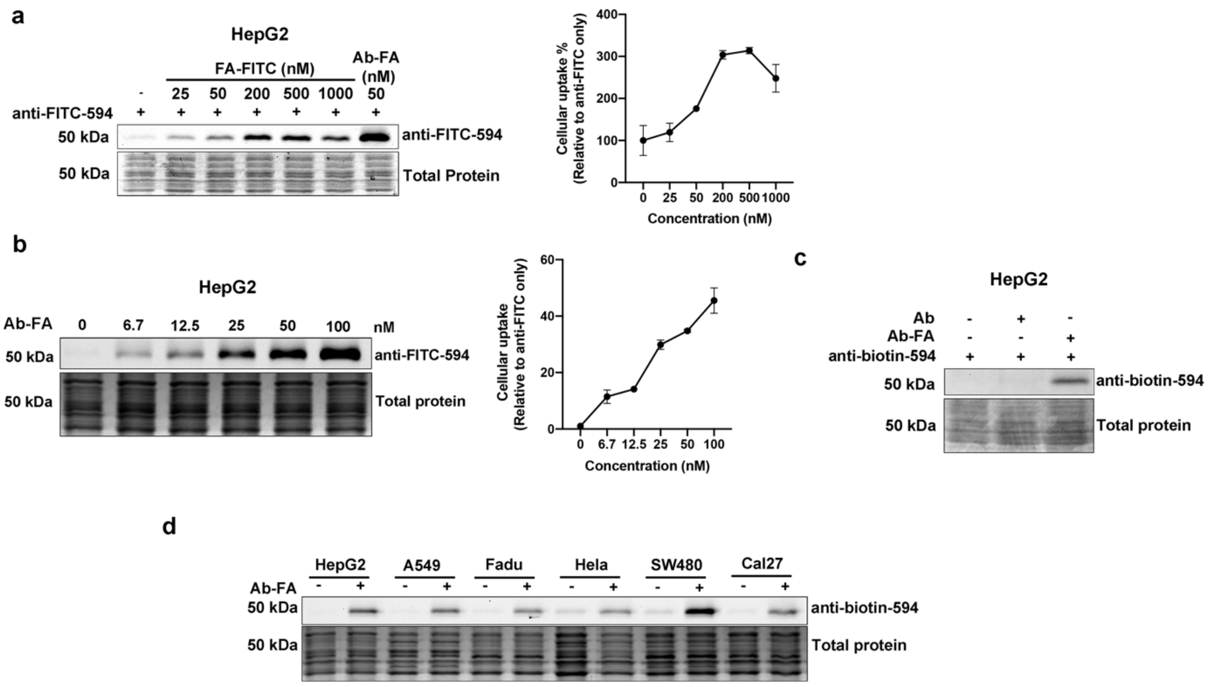


**Supplementary Figure 1: Uptake of soluble protein targets mediated by FRTACs is dose- and cell line-dependent. a.** Dose response of anti-FITC-594 (50 nM) uptake induced by FA-FITC and comparison with 50 nM Ab-FA in HepG2 cells for 3 h (n = 3). **B.** Dose response of anti-FITC-594 (50 nM) uptake induced by Ab-FA in HepG2 cells for 3 h (n = 3). **c.** Uptake of anti-biotin-594 (50 nM) in HepG2 cells treated with Ab-FA (25 nM) for 3 h. **d.** Comparison of anti-biotin-594 (50 nM) uptake in different cancer cell lines treated with Ab-FA (25 nM) for 24 h. N indicates biologically independent experiments. Data are presented as mean ± SD. Source data are provided as a Source Data file.

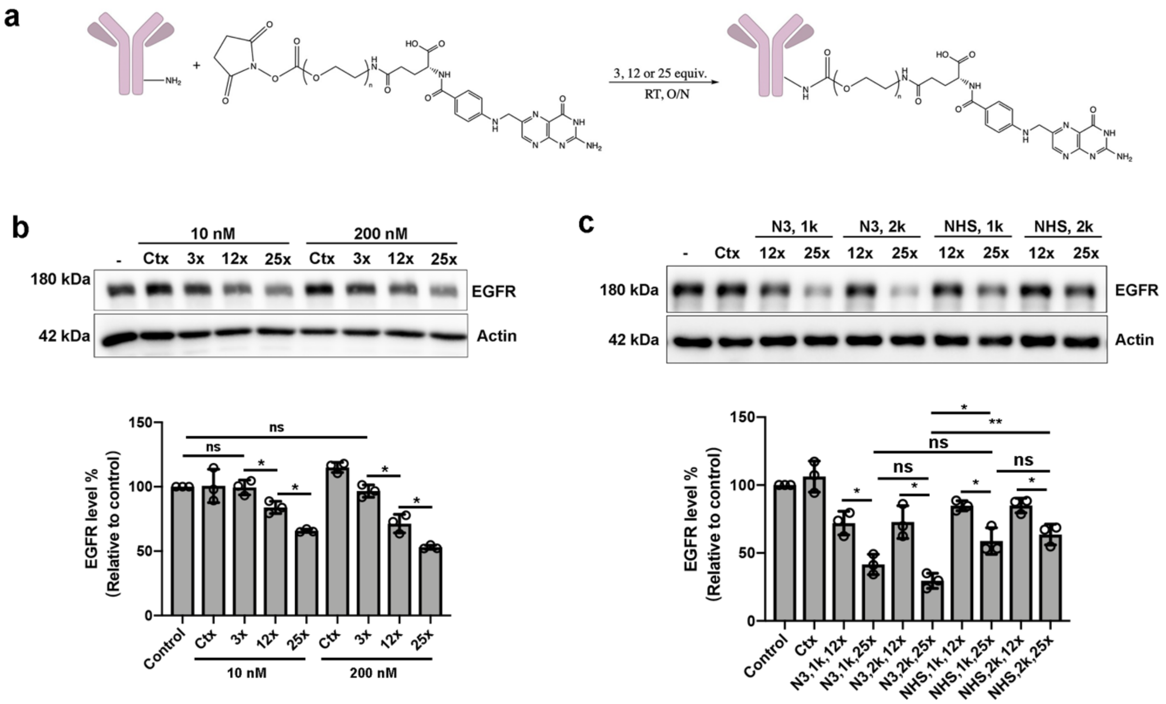


**Supplementary Figure 2: FRTACs generated by two-step labeling method with more FA labeling and longer linker length have higher protein degradation efficiency. a.** Generation of FRTAC by one-step labeling method. **b.** EGFR degradation mediated by FRTACs prepared by one-step labeling method using different amounts of folate-NHS ester (3x, 12x, and 25x) (n = 3). **c.** Comparison of EGFR degradation induced by FRTACs prepared by one- or two-step labeling method with various amounts of reagents (n = 3). 3x: 3 molar equivalents, 12x: 12 molar equivalents, 25x: 25 molar equivalents, 1k: PEG1k linker, 2k: PEG2k linker. N3: two-step labeling (12x and 25x indicate the equivalence of the DBCO-NHS ester in the first step; 25 equivalents of folate-azide was used in the second step). NHS: one-step labelling. N indicates biologically independent experiments. Data are presented as mean ± SD. The statistical significance was assessed using an unpaired two-tailed t-test, *P < 0.05, **P < 0.01, ns: not significant. Source data are provided as a Source Data file.

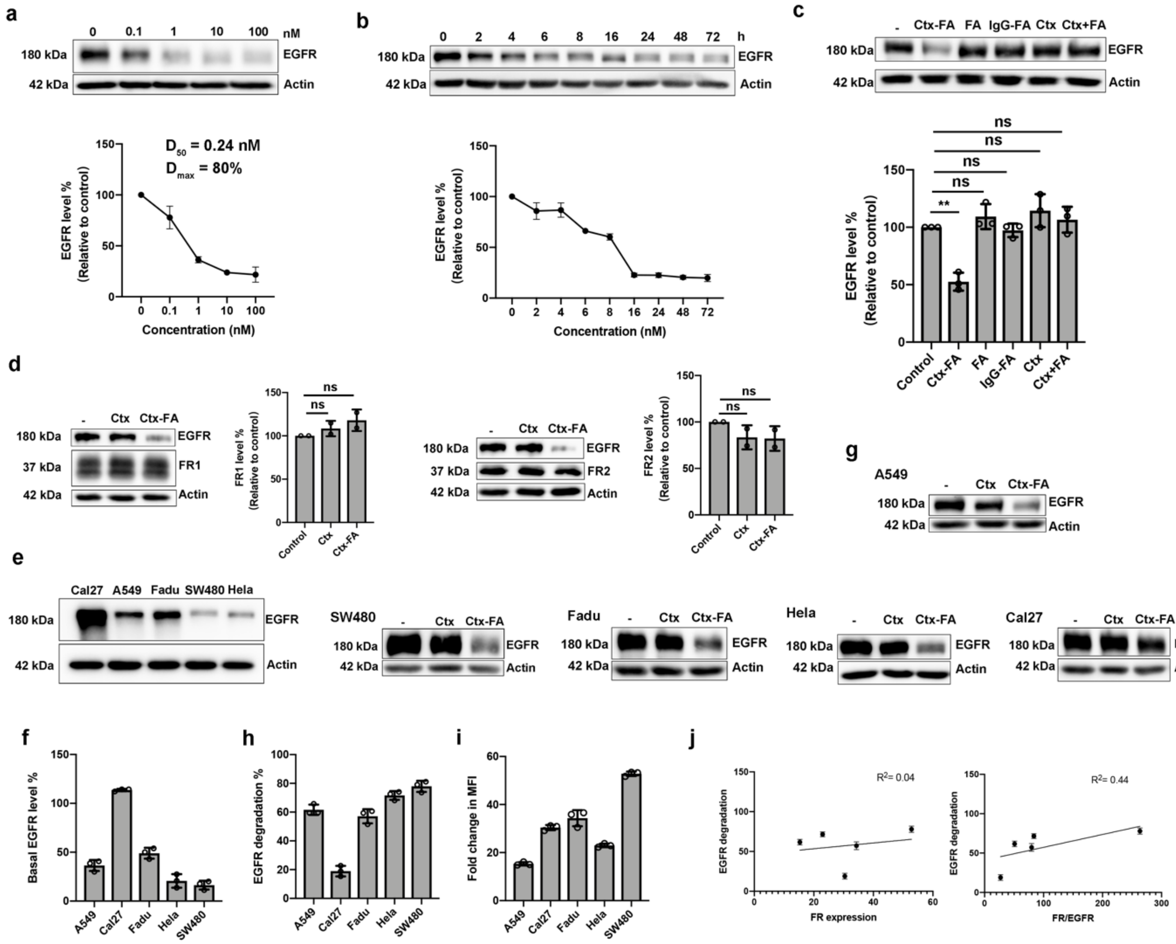

**Supplementary Figure 3: EGFR degradation is related to both FR and EGFR expression levels. a.** Dose response of EGFR degradation mediated by Ctx-FA (24 h) in Hela cell (n = 3). **b.** Time course of EGFR degradation mediated by Ctx-FA (10 nM) in Hela cell (n = 3). **c.** EGFR degradation mediated by Ctx-FA and negative controls (n = 3). **d.** FR1 and FR2 level before and after degrader treatment (n = 2). **e.** Endogenous EGFR expression level in different cancer cell lines. **f.** Quantification of **e (**n = 3)**. g.** EGFR degradation in different cancer cell lines. **h.** Quantification of **g (**n = 3)**. i.** Quantification of FR expression levels on different cancer cell lines by flow cytometry (n = 3). **j.** Correlation of EGFR degradation efficiency with FR expression level alone or the ratio of FR and endogenous EGFR on different cancer cell lines. N indicates biologically independent experiments. Data are presented as mean ± SD. The statistical significance was assessed using an unpaired two-tailed t-test, **P < 0.01, ns: not significant. Source data are provided as a Source Data file.

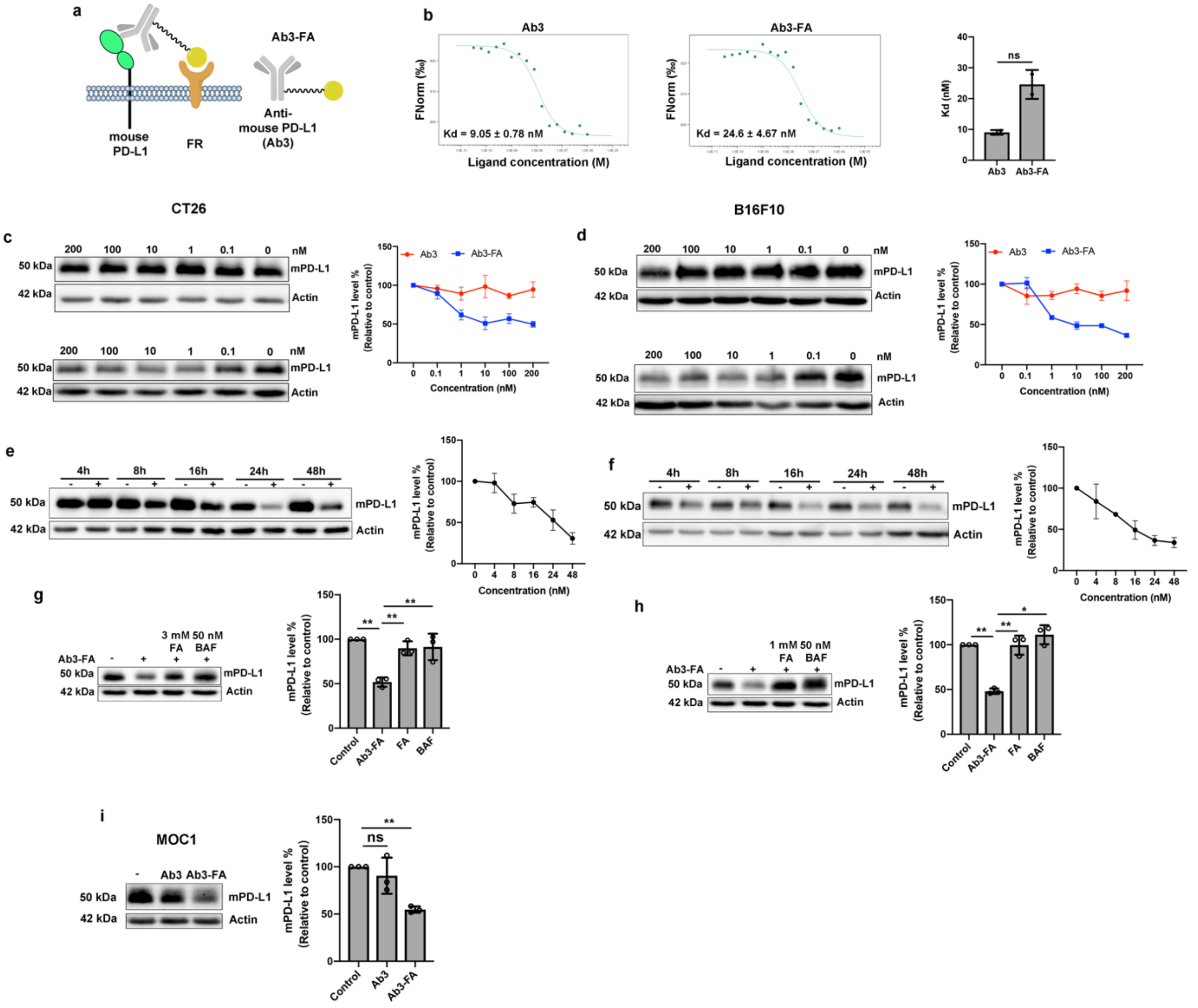


**Supplementary Figure 4: FRTACs mediate the lysosomal degradation of mPD-L1 via their interaction with FR in three mouse cell lines (CT26: c, e, g; B16F10: d, f, h; MOC1: i). a.** Schematic of Ab3-FA targeting mouse PD-L1 (mPD-L1). **b.** Representative plot of the binding affinity of Ab3 and Ab3-FA to mPD-L1 characterized by MicroScale Thermophoresis (MST) (n = 2). **c, d.** Dose response of mPD-L1 degradation (24 h) mediated by Ab3 and Ab3-FA (n = 3). **e, f.** Time course of mPD-L1 degradation mediated by Ab3-FA (10 nM) (n = 3). **g, h.** Inhibition of Ab3-FA (10 nM) mediated mPD-L1 degradation by free FA (3 mM for CT26, 1 mM for B16F10) and Bafilomycin A1 (BAF, 50 nM) for 24 h (n = 3). **i.** Cellular mPD-L1 degradation in MOC1 cells mediated by Ab3-FA (10 nM, 24 h) (n = 3). N indicates biologically independent experiments. Data are presented as mean ± SD. The statistical significance was assessed using an unpaired two-tailed t-test, *P < 0.05, **P < 0.01, ns: not significant. Source data are provided as a Source Data file.

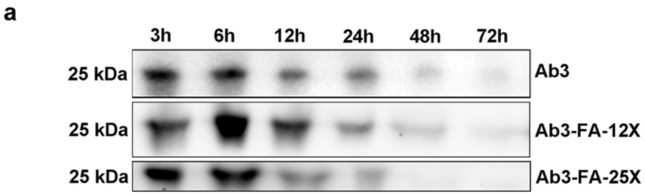


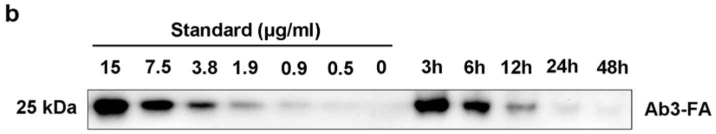


**Supplementary Figure 5: Evaluation of the pharmacokinetics (PK) for PD-L1 degraders *in-vivo*. a.** Representative blot of rat IgG in the plasma of C57BL/6 mice treated with Ab3, Ab3-FA-12x, Ab3-FA-25x at different time points (2.5 mg/kg via IP injection) from 4 mice. **b.** Representative blot of rat IgG in the plasma of C57BL/6 mice bearing B16F10 tumor treated with Ab3-FA-25x at different time points (2.5 mg/kg via IP injection) from 3 mice. Ab3-FA-25x at different concentrations were used as standard. (12x and 25x: 12 or 25 molar equivalents of DBCO-NHS ester in the first step; 25 equivalents of folate-azide were used in the second step). Source data are provided as a Source Data file.

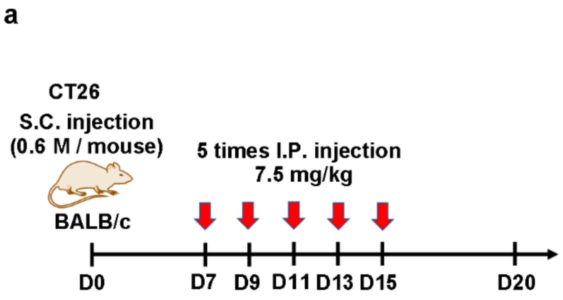


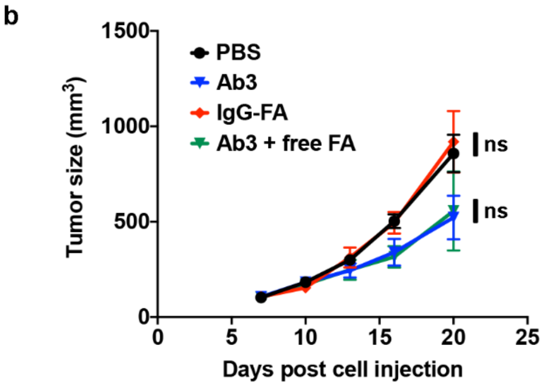


**Supplementary Figure 6: Non-targeting FA conjugates and free FA have no effects on tumor growth in CT26 syngeneic mouse model. a.** Schematic illustration of control treatment in CT26 mouse model. **b.** Tumor growth curves after different treatments as indicated by a. Data are presented as mean ± SD, n = 6 mice. The statistical significance was assessed using a paired t-test, ns: not significant. Source data are provided as a Source Data file.

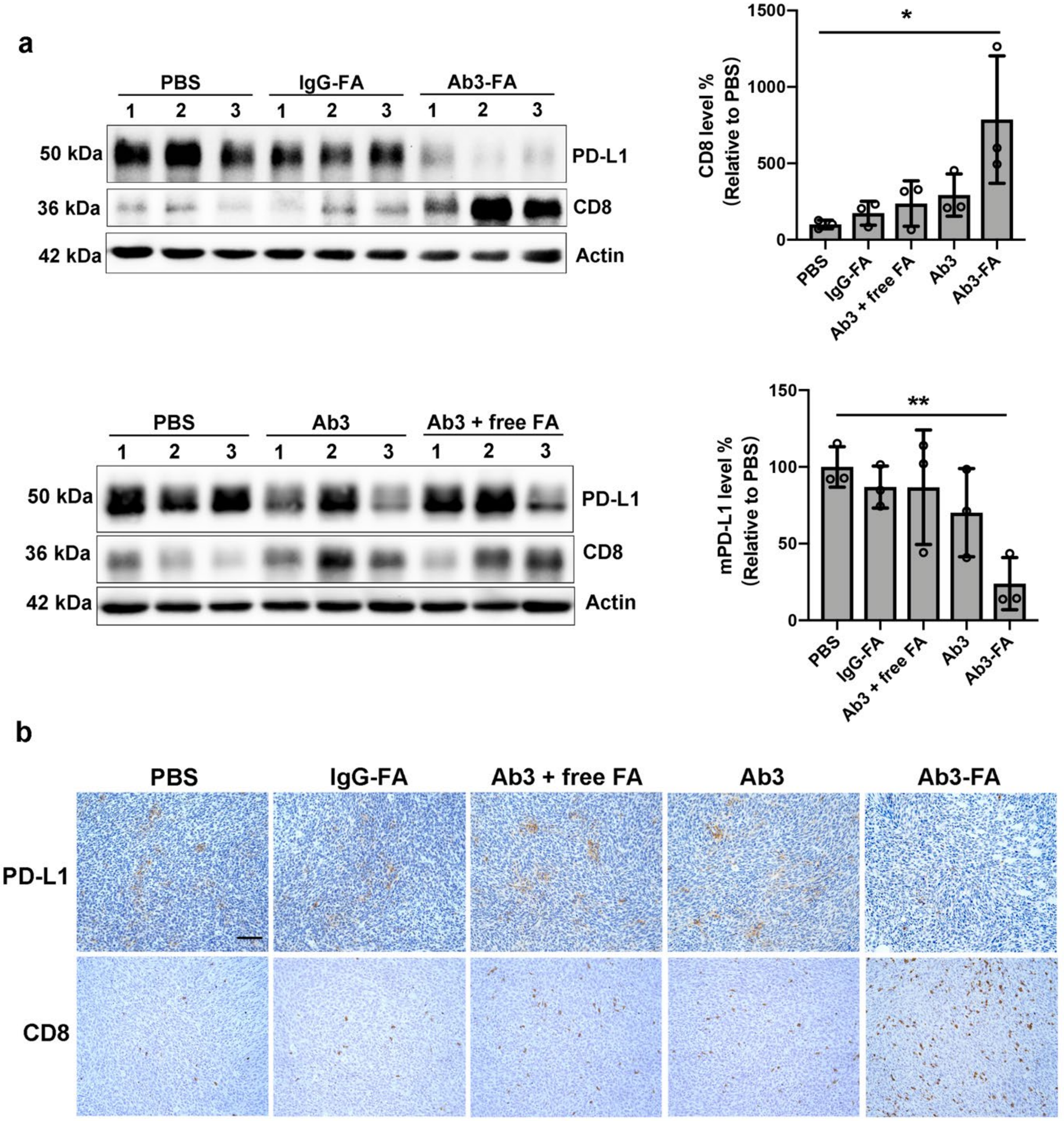


**Supplementary Figure 7: FRTAC reveals anti-tumor effect by degrading PD-L1 and recruiting cytotoxic T cell into the tumor. A.** Western blot analysis of PD-L1 and CD8a level in tumor tissues isolated from CT26 tumor-bearing mice (7.5 mg/kg daily for 3 days, 3 mice/cohort). **B.** Detection of PD-L1 and CD8 in CT26 tumors by IHC staining (7.5 mg/kg daily for 3 days, 3 mice/cohort). Scale bar: 100 µm**.** Representative figures from three mice. Data are presented as mean ± SD, n=3 mice. The statistical significance was assessed using an unpaired two-tailed t-test, *P < 0.05, **P < 0.01. Source data are provided as a Source Data file.

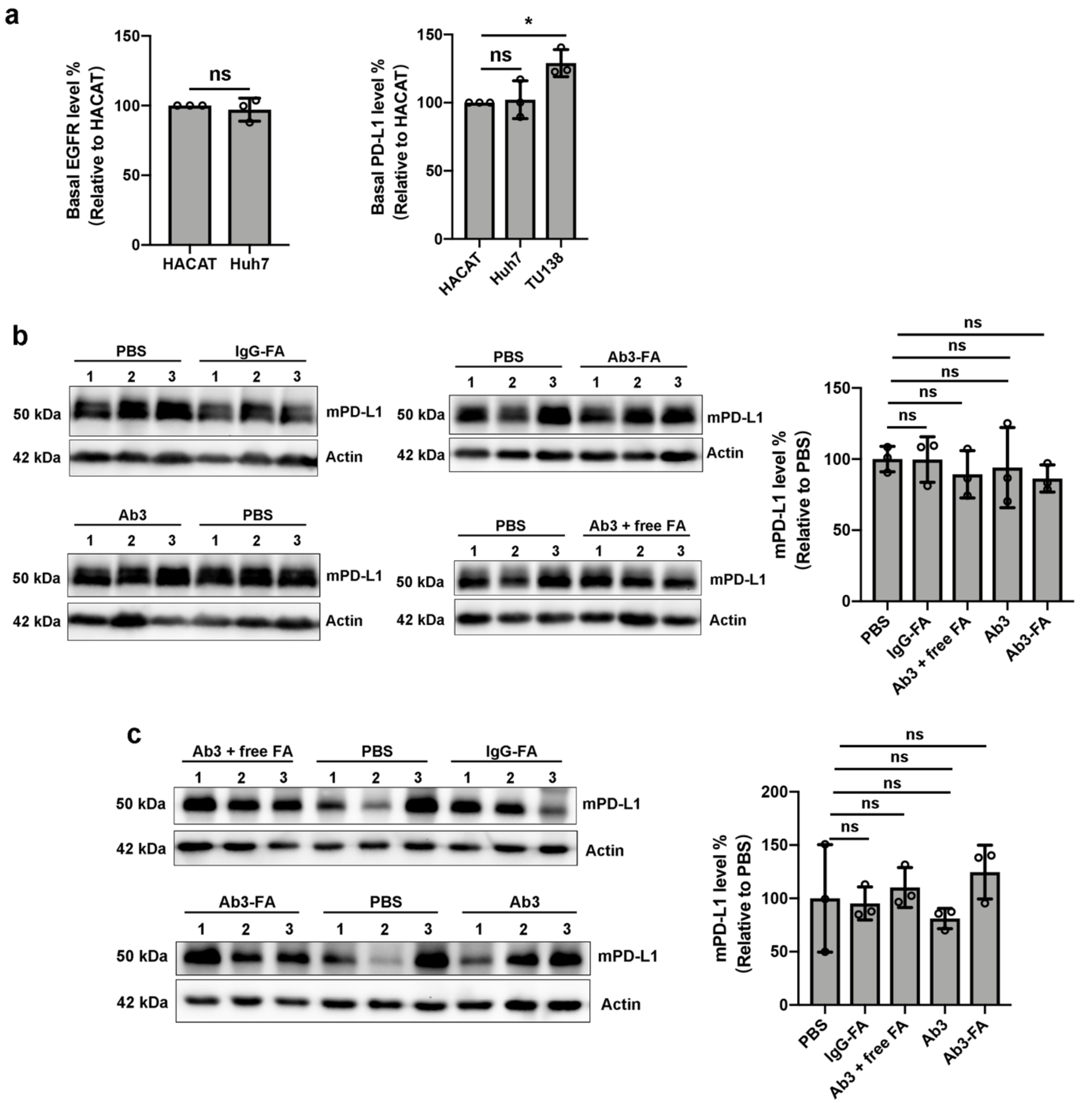


**Supplementary Figure 8: FRTACs exhibit cancer selectivity both *in-vitro* and *in-vivo*. a.** Basal levels of EGFR and PD-L1 in normal HACAT and cancer cell line Huh7 and TU138 (n = 3 biologically independent experiments). **b.** Mouse PD-L1 level in spleen, n = 3 mice. **c.** Mouse PD-L1 level in lung, n = 3 mice. Data are presented as mean ± SD. The statistical significance was assessed using an unpaired two-tailed t-test, *P < 0.05, ns: not significant. Source data are provided as a Source Data file.

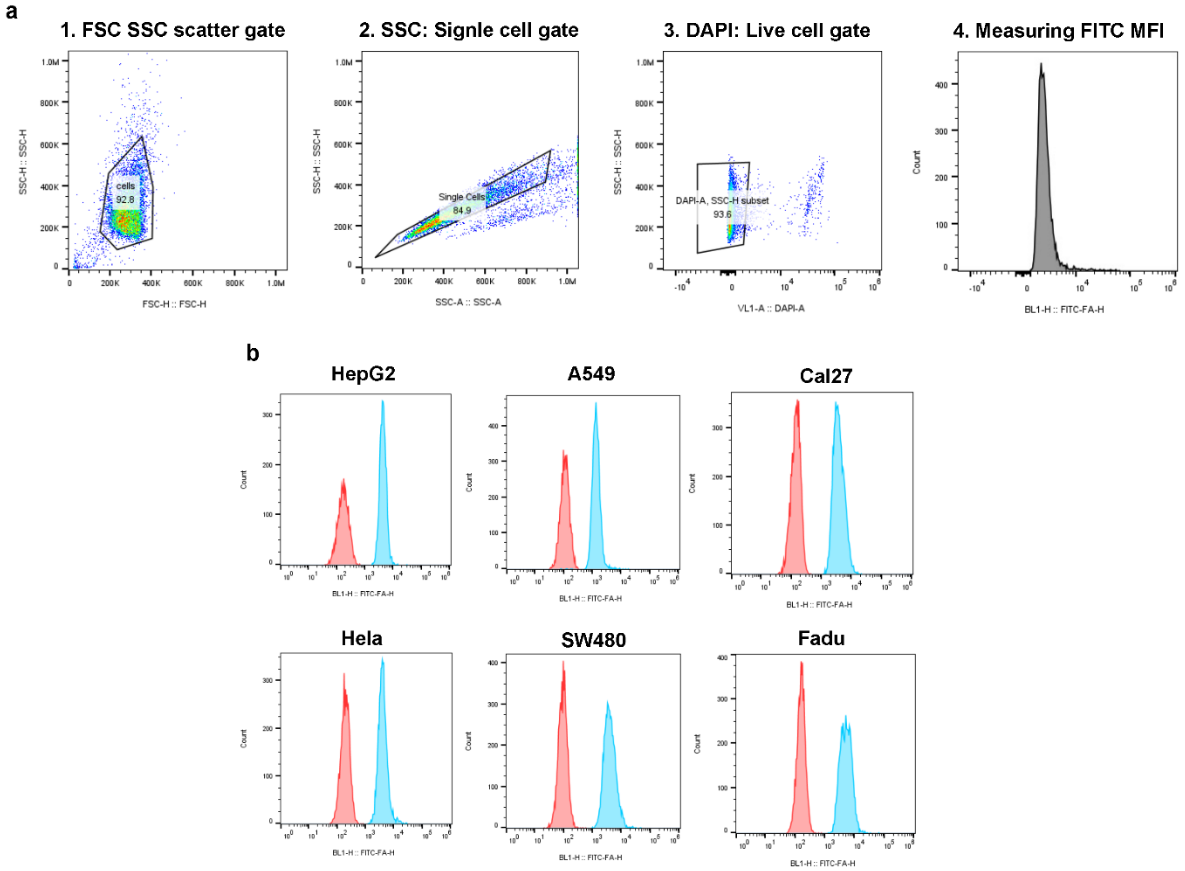


**Supplementary Figure 9. Characterization of FR expresstion levels on different cell lines by flow cytometry. a.** Gating strategies for flow cytometry analysis presented in b, figure 2g and Supplementary Figure 3i. **b.** Representative flow cytometry histogram of FR expression level on different cancer cell lines. n = 10,000 live cells analyzed over three biologically independent experiments.

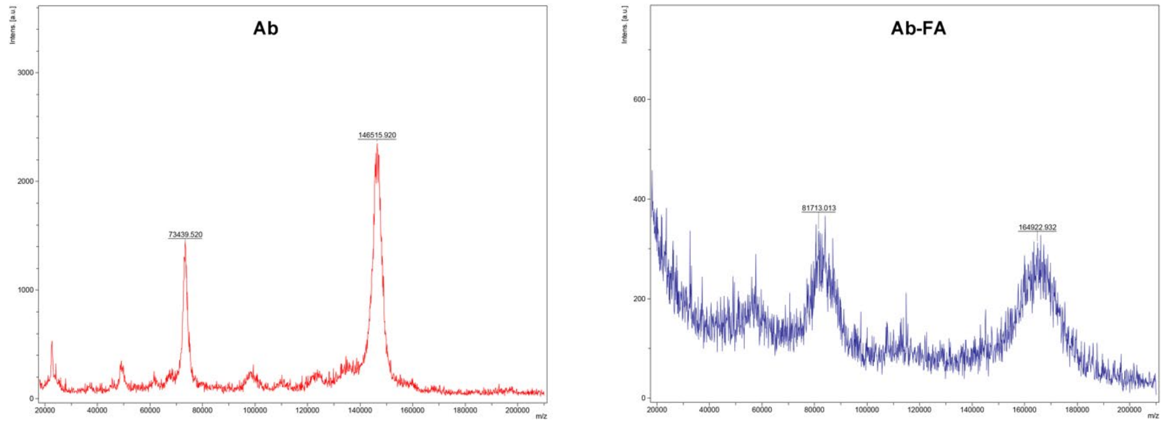


**Supplementary Figure 10a. MALDI-TOF-MS Characterization of Ab (anti-mouse IgG) -FA.**

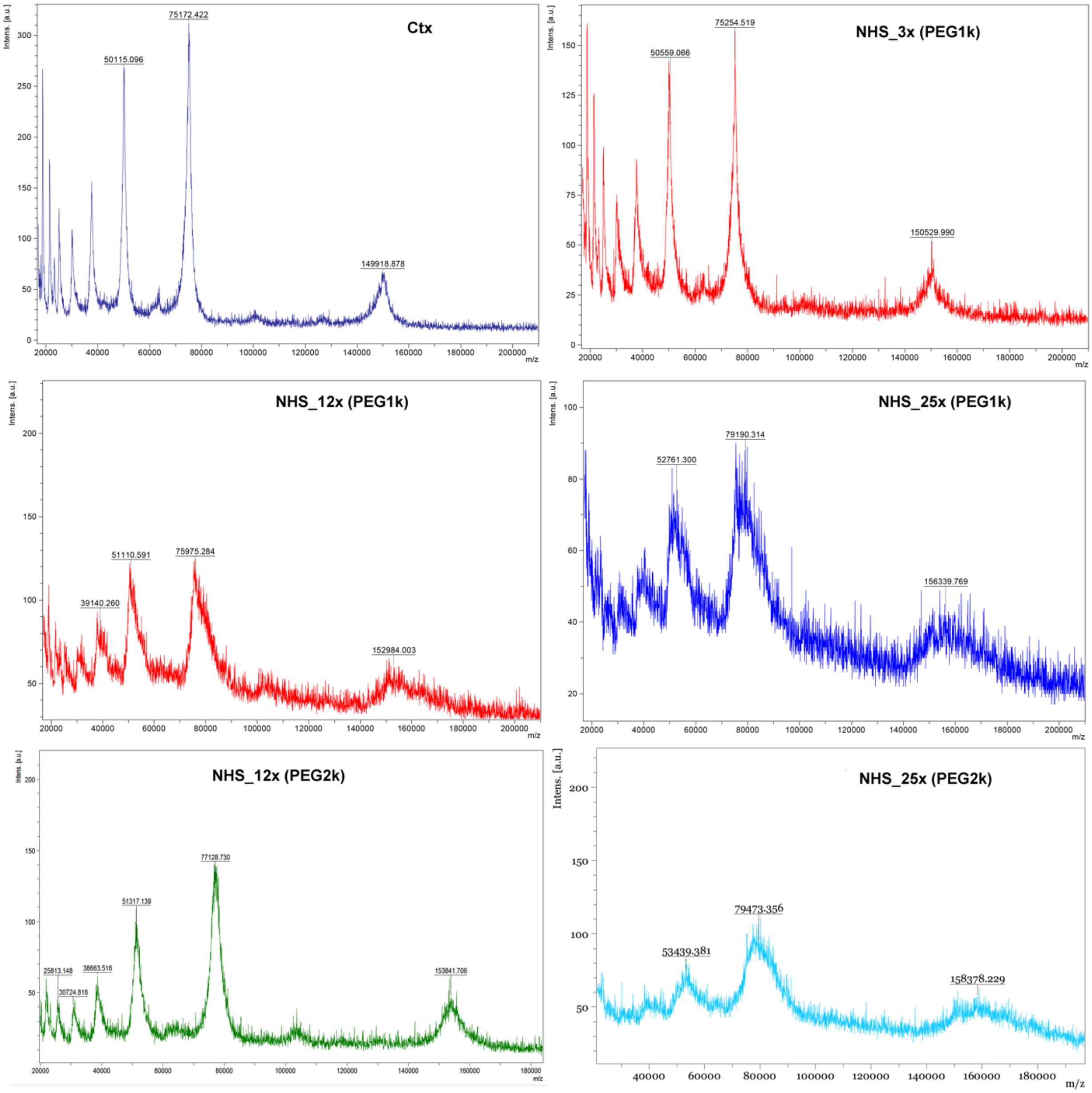


**Supplementary Figure 10b. MALDI-TOF-MS Characterization of Ctx-FA generated by one-step labeling.**

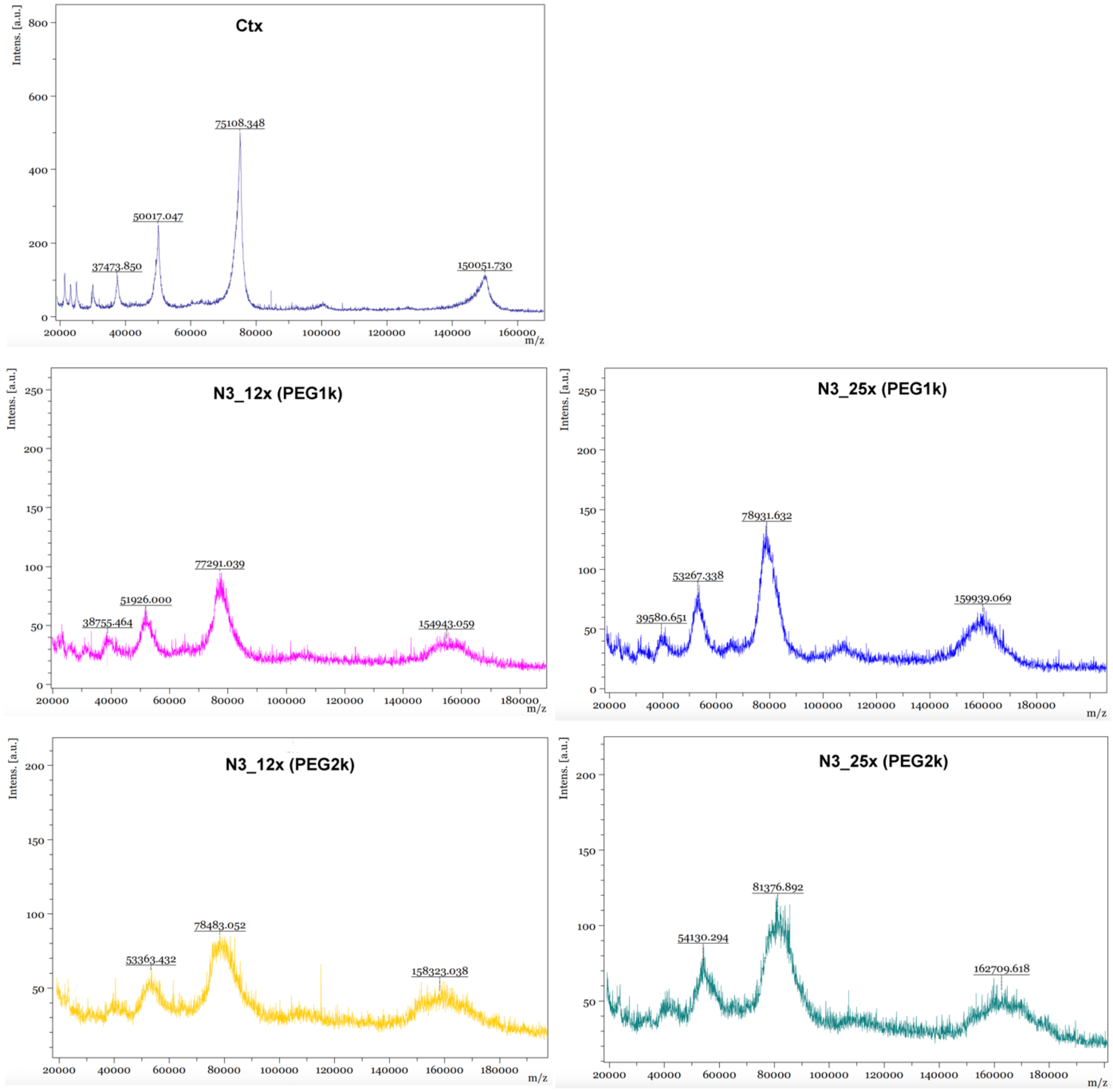


**Supplementary Figure 10c. MALDI-TOF-MS Characterization of Ctx-FA generated by two-step labeling.**

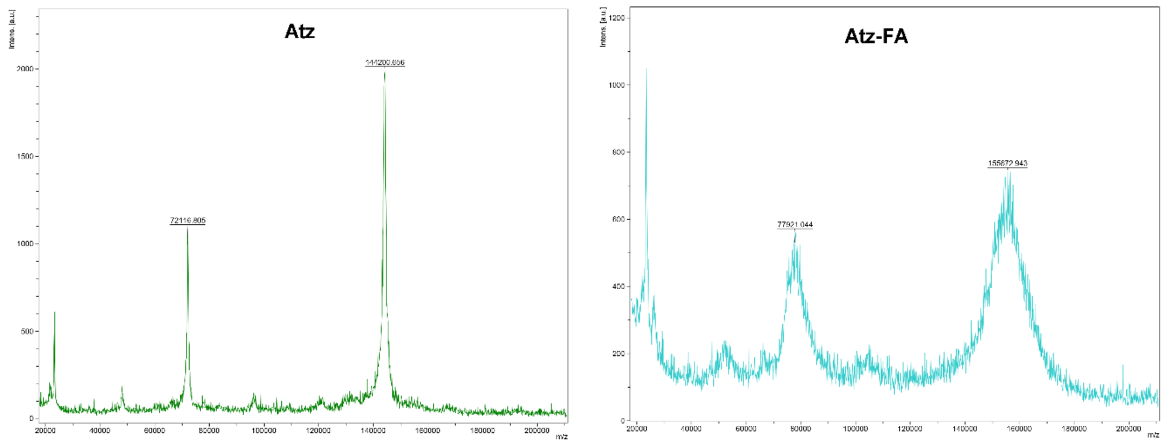


**Supplementary Figure 10d. MALDI-TOF-MS Characterization of Atz-FA.**

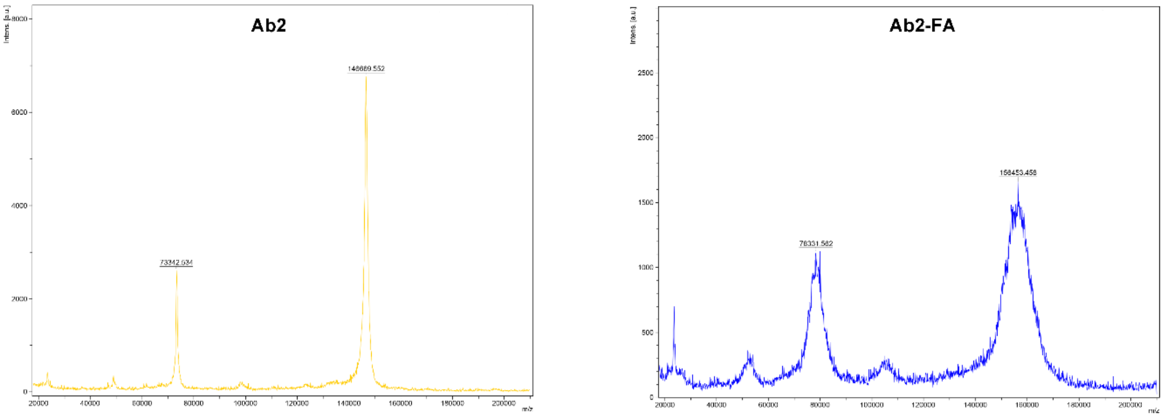


**Supplementary Figure 10e. MALDI-TOF-MS Characterization of Ab2 (anti-CD47)-FA.**

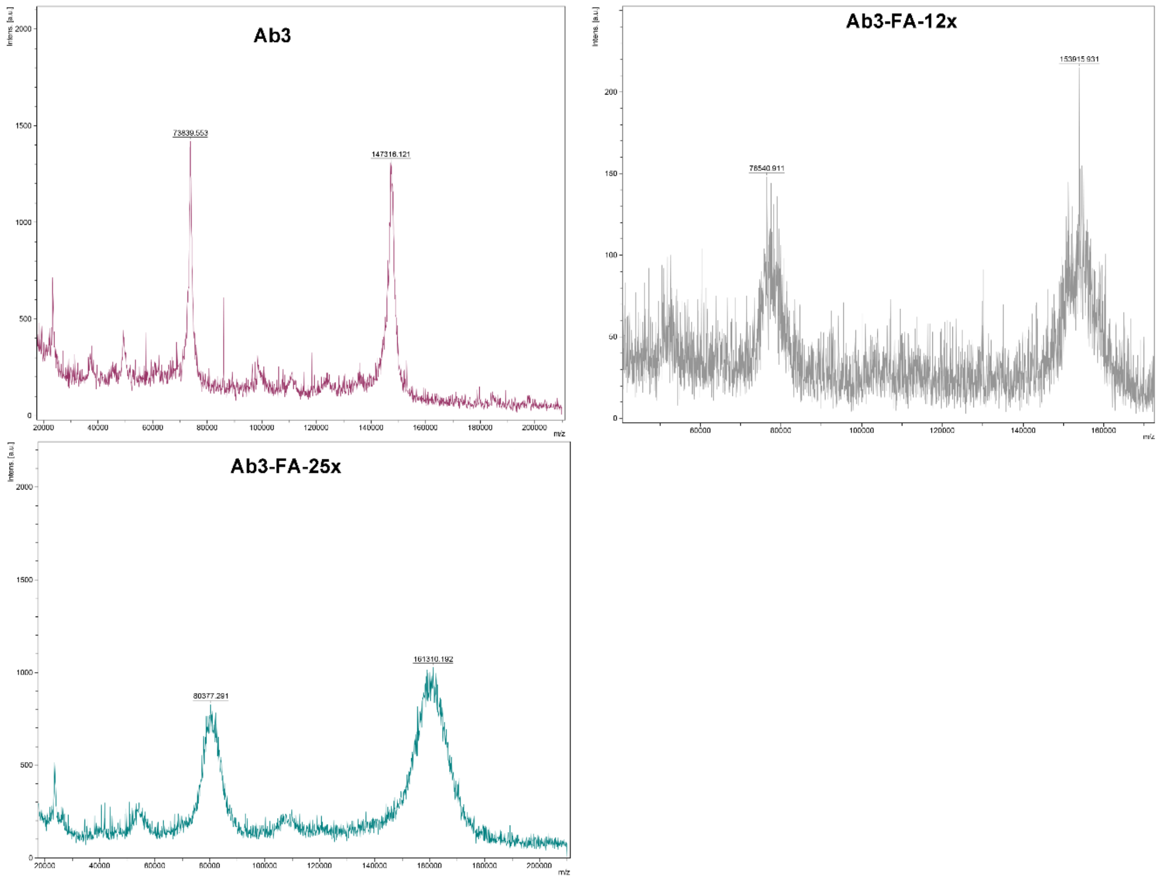


**Supplementary Figure 10f. MALDI-TOF-MS Characterization of Ab3 (anti-mouse PD-L1)-FA.**

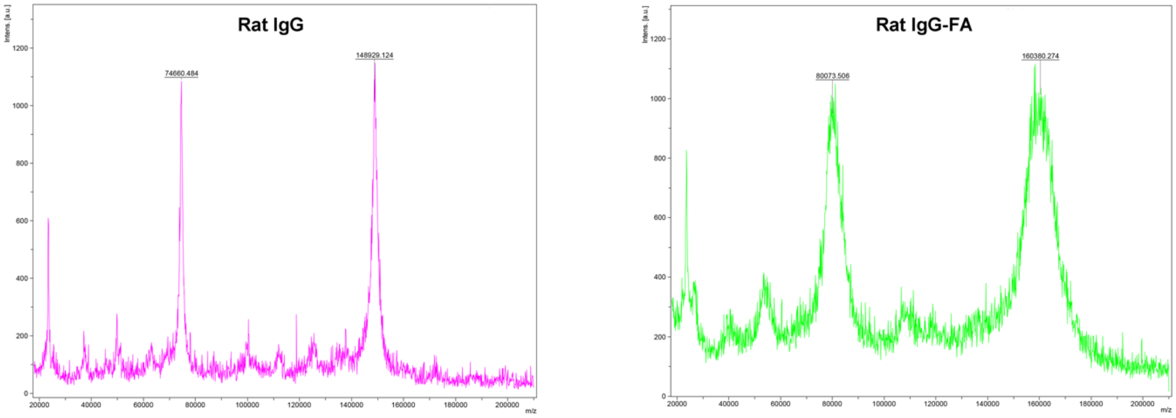


**Supplementary Figure 10g. MALDI-TOF-MS Characterization of Rat IgG-FA.**

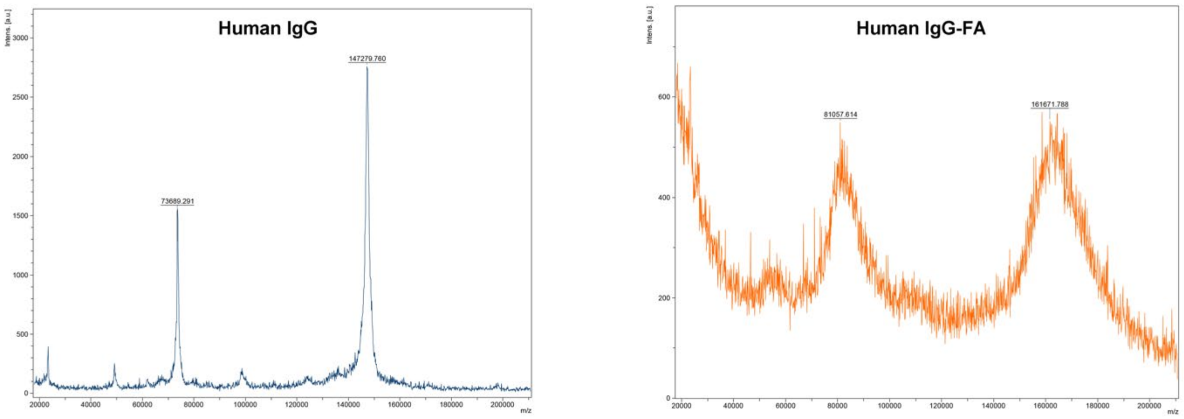


**Supplementary Figure 10h. MALDI-TOF-MS Characterization of Human IgG-FA.**

**Supplementary Table 1. Sources of key reagents and antibodies**

| Reagents and antibodies | Vendor | Catalog # |
|---|---|---|
| DBCO-PEG3-$CH_2$CO-NHS ester | Axis | AP10030 |
| Folic acid-PEG1k-NHS* | Ruixibio | R-1112 |
| Folic acid-PEG2k-NHS* | Ruixibio | R-1112 |
| Folic acid-PEG1k-$N_3$* | Ruixibio | R-8867 |
| Folic acid-PEG2k-$N_3$* | Ruixibio | R-8867 |
| Folic acid-PEG2k-FITC* | Ruixibio | R-8861 |
| AffiniPure Goat Anti-Mouse IgG | Jackson ImmunoResearch | 115-005-062 |
| Alexa Fluor® 594 IgG Fraction Monoclonal Mouse Anti-Fluorescein (FITC) | Jackson ImmunoResearch | 200-582-037 |
| Anti-Biotin Mouse Monoclonal Antibody (Alexa Fluor®647) | Jackson ImmunoResearch | 200-602-211 |
| Anti-Biotin Mouse Monoclonal Antibody (Alexa Fluor®594) | Jackson ImmunoResearch | 200-582-211 |
| Mouse Anti-Rabbit IgG Antibody (Alexa Fluor® 647) | Jackson ImmunoResearch | 211-605-109 |
| Cetuximab | Selleckchem | A2000 |
| Atezolizumab | Selleckchem | A2004 |
| *InVivo*Plus anti-mouse PD-L1 (B7-H1) | Bioxcell | BP0101 |
| *InVivo*MAb anti-human CD47 | Bioxcell | BE0019-1 |
| *InVivo*Plus rat IgG2b isotype control | Bioxcell | BP0090 |
| *InVivo*MAb human IgG1 isotype control | Bioxcell | BE0297 |
| Rab7 (D95F2) XP® Rabbit mAb | Cell Signaling Technology | #9367s |
| EGF Receptor (D38B1) XP® Rabbit mAb | Cell Signaling Technology | #4267s |
| LAMP1 (D4O1S) Mouse mAb | Cell Signaling Technology | #15665s |
| DYKDDDDK Tag (D6W5B) Rabbit mAb | Cell Signaling Technology | #147935s |
| beta Actin Antibody (C4) | Santa Cruz | sc-47778 |
| Phospho-p44/42 MAPK (Erk1/2) (Thr202/Tyr204) (E10) Mouse mAb | Cell Signaling Technology | #9106s |
| Phospho-EGF Receptor (Tyr1068) (D7A5) XP® Rabbit mAb | Cell Signaling Technology | #3777s |

| | | |
|---|---|---|
| PD-L1 (E1L3N®) XP® Rabbit mAb | Cell Signaling Technology | #13684s |
| Recombinant Anti-CD47 antibody | Abcam | ab218810 |
| PD-L1 (D4H1Z) Rabbit mAb | Cell Signaling Technology | 60475s |
| Peroxidase AffiniPure Goat Anti-Rat IgG (H+L) | Jackson ImmunoResearch | 112-035-167 |
| PD-L1 (D5V3B) Rabbit mAb | Cell Signaling Technology | #64988s |
| PD-L1/CD274 Polyclonal antibody | Proteintech | 17952-1-AP |
| CD8α (D4W2Z) XP® Rabbit mAb | Cell Signaling Technology | #98941s |
| FOLR1 Polyclonal antibody | Proteintech | 23355-1-AP |
| FR Antibody (E-11) | Santa Cruz Biotechnology | sc-515521 |
| FOLR2 Polyclonal antibody | Invitrogen | PA5-45768 |
| Goat anti-Mouse IgG (H+L) Alexa Fluor 488 | Invitrogen | A11001 |
| Goat anti-Rabbit IgG (H+L) Cross-Adsorbed Secondary Antibody, Alexa Fluor™ 594 | Invitrogen | A-11012 |
| Anti-rabbit IgG, HRP-linked Antibody | Cell Signaling Technology | #7074s |
| Anti-mouse IgG, HRP-linked Antibody | Cell Signaling Technology | #7076s |
| ImmPRESS® HRP Goat Anti-Mouse IgG Polymer Detection Kit, Peroxidase | Vector Laboratories | MP-7452 |
| ImmPRESS® HRP Goat Anti-Rabbit IgG Polymer Detection Kit, Peroxidase | Vector Laboratories | MP-7451 |
| DAPI Solution (1mg/mL) | Thermo Fisher Scientific | 62248 |
| Chloroquine diphosphate salt | Sigma-Aldrich | C6628-25G |
| Bafilomycin A1 Ready Made Solution | Sigma-Aldrich | SML1661 |
| MG132 | Selleckchem | S2619 |
| Methyl-β-cyclodextrin | Thermo Fisher Scientific | J66847.06 |
| Chlorpromazine | Thermo Fisher Scientific | J63659.09 |
| Cytochalasin D | Invitrogen | PHZ1063 |
| Folic acid | Sigma-Aldrich | F7876 |

| | | |
|---|---|---|
| FOLR1 cDNA ORF Clone, Human, N-DYKDDDDK (Flag®) tag | SinoBiological | HG11241-NF |
| FOLR2 cDNA ORF Clone, Human, N-DYKDDDDK (Flag®) tag | SinoBiological | HG11219-NF |
| ON-TARGETplus Human FOLR1 siRNA | Dharmacon | L-010403-00-0005 |
| ON-TARGETplus Human RAB7A siRNA | Dharmacon | L-010388-00-0005 |
| Lipofectamine™ RNAiMAX Transfection Reagent | Thermo Fisher Scientific | 13778075 |

* 1k and 2k refer to the average molecular weight of the PEG (polyethylene glycol) linker.

**Supplementary Table 2. Fold change of mean fluorescent intensity (MFI) of FR expression level measured by flow cytometry**

| | MFI | | |
|---|---|---|---|
| | unstained | stained | fold change |
| HepG2 | 231 | 6709 | 29.04 |
| A549 | 145 | 2175 | 15.00 |
| Cal27 | 181 | 5644 | 31.18 |
| Hela | 304 | 6772 | 22.28 |
| SW480 | 116 | 6199 | 53.44 |
| Fadu | 239 | 8747 | 36.60 |

## Supplementary Table 3. Average number of FA labeled on the FRTACs

| | Ave. No of FA per antibody |
|---|---|
| Ab-FA | 6.26 |
| NHS_3x (PEG1k) | 0.27 |
| NHS_12x (PEG1k) | 2.1 |
| NHS_25x (PEG1k) | 5.02 |
| NHS_12x (PEG2k) | 2.46 |
| NHS_25x (PEG2k) | 4.26 |
| N3_12x (PEG1k) | 2.89 |
| N3_25x (PEG1k) | 5.48 |
| N3_12x (PEG2k) | 2.89 |
| N3_25x (PEG2k) | 4.85 |
| Atz-FA | 4.72 |
| Ab2-FA | 3.59 |
| Ab3-FA-12x | 2.27 |
| Ab3-FA-25x | 5.02 |
| Rat IgG-FA | 3.78 |
| Human IgG-FA | 5.33 |
