# Supplementary material for: Development of folate receptor targeting chimeras for cancer selective degradation of extracellular proteins
Source: Nat Commun. 2024 Oct 8;15:8695. doi: 10.1038/s41467-024-52685-9 (PMC11461649; doi:10.1038/s41467-024-52685-9)
Supplement: Supplementary file 3 — Reporting Summary [file 41467_2024_52685_MOESM3_ESM.pdf]

nature portfolio

Corresponding author(s): Weiping Tang

Last updated by author(s): Sep 9, 2024

Reporting Summary

Nature Portfolio wishes to improve the reproducibility of the work that we publish. This form provides structure for consistency and transparency in reporting. For further information on Nature Portfolio policies, see our Editorial Policies and the Editorial Policy Checklist.

Statistics

For all statistical analyses, confirm that the following items are present in the figure legend, table legend, main text, or Methods section.

n/a	Confirmed	
☐	☒	The exact sample size (n) for each experimental group/condition, given as a discrete number and unit of measurement
☐	☒	A statement on whether measurements were taken from distinct samples or whether the same sample was measured repeatedly
☐	☒	The statistical test(s) used AND whether they are one- or two-sided<br>*Only common tests should be described solely by name; describe more complex techniques in the Methods section.*
☒	☐	A description of all covariates tested
☒	☐	A description of any assumptions or corrections, such as tests of normality and adjustment for multiple comparisons
☐	☒	A full description of the statistical parameters including central tendency (e.g. means) or other basic estimates (e.g. regression coefficient) AND variation (e.g. standard deviation) or associated estimates of uncertainty (e.g. confidence intervals)
☐	☒	For null hypothesis testing, the test statistic (e.g. F, t, r) with confidence intervals, effect sizes, degrees of freedom and P value noted<br>*Give P values as exact values whenever suitable.*
☒	☐	For Bayesian analysis, information on the choice of priors and Markov chain Monte Carlo settings
☒	☐	For hierarchical and complex designs, identification of the appropriate level for tests and full reporting of outcomes
☐	☒	Estimates of effect sizes (e.g. Cohen's d, Pearson's r), indicating how they were calculated

Our web collection on statistics for biologists contains articles on many of the points above.

Software and code

Policy information about availability of computer code

Data collection	MALDI-MS plots were generated by Bruker flexAnalysis (v 4.2). Western blot and in-gel fluorescence images were acquired by Image Lab Touch Software (v 2.0.1.35). Immunofluorescent images were acquired by Leica Application Suite X (LAS X v2.6) software. Flow cytometry data were acquired using ThermoFisher Attune NxT cytometric software (v 6).
Data analysis	Western blot bands and Immunofluorescent images were analyzed using ImageJ (v 1.53a). Graphpad Prism 9 was used for data analysis. FlowJo (v 10.8.1) was used for flow cytometry data analysis. Immunofluorescent images were analyzed by Leica Application Suite X (LAS X v2.6) software.

For manuscripts utilizing custom algorithms or software that are central to the research but not yet described in published literature, software must be made available to editors and reviewers. We strongly encourage code deposition in a community repository (e.g. GitHub). See the Nature Portfolio guidelines for submitting code & software for further information.

Data

Policy information about availability of data

All manuscripts must include a data availability statement. This statement should provide the following information, where applicable:

- Accession codes, unique identifiers, or web links for publicly available datasets
- A description of any restrictions on data availability
- For clinical datasets or third party data, please ensure that the statement adheres to our policy

All data presented in this study are included in the manuscript and Supplementary Information. Source data are provided with this paper.

Research involving human participants, their data, or biological material

Policy information about studies with human participants or human data. See also policy information about sex, gender (identity/presentation), and sexual orientation and race, ethnicity and racism.

Reporting on sex and gender	*Use the terms sex (biological attribute) and gender (shaped by social and cultural circumstances) carefully in order to avoid confusing both terms. Indicate if findings apply to only one sex or gender; describe whether sex and gender were considered in study design; whether sex and/or gender was determined based on self-reporting or assigned and methods used. Provide in the source data disaggregated sex and gender data, where this information has been collected, and if consent has been obtained for sharing of individual-level data; provide overall numbers in this Reporting Summary. Please state if this information has not been collected. Report sex- and gender-based analyses where performed, justify reasons for lack of sex- and gender-based analysis.*
Reporting on race, ethnicity, or other socially relevant groupings	*Please specify the socially constructed or socially relevant categorization variable(s) used in your manuscript and explain why they were used. Please note that such variables should not be used as proxies for other socially constructed/relevant variables (for example, race or ethnicity should not be used as a proxy for socioeconomic status). Provide clear definitions of the relevant terms used, how they were provided (by the participants/respondents, the researchers, or third parties), and the method(s) used to classify people into the different categories (e.g. self-report, census or administrative data, social media data, etc.) Please provide details about how you controlled for confounding variables in your analyses.*
Population characteristics	*Describe the covariate-relevant population characteristics of the human research participants (e.g. age, genotypic information, past and current diagnosis and treatment categories). If you filled out the behavioural & social sciences study design questions and have nothing to add here, write "See above."*
Recruitment	*Describe how participants were recruited. Outline any potential self-selection bias or other biases that may be present and how these are likely to impact results.*
Ethics oversight	*Identify the organization(s) that approved the study protocol.*

Note that full information on the approval of the study protocol must also be provided in the manuscript.

Field-specific reporting

Please select the one below that is the best fit for your research. If you are not sure, read the appropriate sections before making your selection.

☒ Life sciences ☐ Behavioural & social sciences ☐ Ecological, evolutionary & environmental sciences

For a reference copy of the document with all sections, see nature.com/documents/nr-reporting-summary-flat.pdf

Life sciences study design

All studies must disclose on these points even when the disclosure is negative.

Sample size	Sample size were determined based on previous lab experience with the variability of different assays (i.e. Westerin blot) and to fulfill statistical analysis requirement. For in-vivo study, the sample sizes were determined to observe statistical difference among different treatment groups. The sizes were indicated in the corresponding figure legends.
Data exclusions	There were no data exclusions from this study.
Replication	Most experiments were repeated three times and such replicates were successful and reproducible, details were provided in the figure legends. For animal studies, the number of mice used in each experiments were provided in the figure legends.
Randomization	Mice were randomly assigned to different treatment groups for in vivo study. For the in vitro experiments, identically cells were seeded and then randomly separated into groups before treatment.
Blinding	MS sample preparation and analysis were conducted by different people. Except for part of the animal study, all other experiments were performed in a non-blinded manner because the authors were responsible for performing the experiments.

Reporting for specific materials, systems and methods

We require information from authors about some types of materials, experimental systems and methods used in many studies. Here, indicate whether each material, system or method listed is relevant to your study. If you are not sure if a list item applies to your research, read the appropriate section before selecting a response.

Materials & experimental systems

n/a	Involved in the study
☐	☒ Antibodies
☐	☒ Eukaryotic cell lines
☐	☐ Palaeontology and archaeology
☐	☒ Animals and other organisms
☐	☐ Clinical data
☐	☐ Dual use research of concern
☐	☐ Plants

Methods

n/a	Involved in the study
☐	☐ ChIP-seq
☐	☒ Flow cytometry
☐	☐ MRI-based neuroimaging

Antibodies

Antibodies used

AffiniPure Goat anti-mouse IgG (115-005-062, Jackson ImmunoResearch, usage varied)
Alexa Fluor® 594 IgG Fraction Monoclonal Mouse Anti-Fluorescein (FITC) (200-582-037, Jackson ImmunoResearch, usage varied)
Anti-Biotin Mouse Monoclonal Antibody (Alexa Fluor®647) (200-602-211, Jackson ImmunoResearch, usage varied)
Anti-Biotin Mouse Monoclonal Antibody (Alexa Fluor®594) (200-582-211, Jackson ImmunoResearch, usage varied)
Mouse Anti-Rabbit IgG Antibody (Alexa Fluor® 647) (211-605-109, Jackson ImmunoResearch, usage varied)
Cetuximab (A2000, Selleckchem, usage varied)
Atezolizumab (A2004, Selleckchem, usage varied)
InVivoPlus anti-mouse PD-L1 (B7-H1, BP0101, Bioxcell, usage varied)
InVivoPlus rat IgG2b isotype control (BP0090, Bioxcell, usage varied)
InVivoMAb human IgG1 isotype control (BE0297,Bioxcell, usage varied)
InVivoMAb anti-human CD47 (BE0019-1, Bioxcell, usage varied)
Rab7 (D95F2) XP® Rabbit mAb (9367s, Cell Signaling Technology, 1:1000)
EGF Receptor (D38B1) XP® Rabbit mAb (4267s, Cell Signaling Technology, 1:1000)
LAMP1 (D4O1S) Mouse mAb (15665s, Cell Signaling Technology, 1:1000)
DYKDDDDK Tag (D6W5B) Rabbit mAb (147935s, Cell Signaling Technology, 1:1000)
beta Actin Antibody (C4) (sc-47778, Santa Cruz, 1:1000)
Phospho-p44/42 MAPK (Erk1/2) (Thr202/Tyr204) (E10) Mouse mAb (9106s, Cell Signaling Technology, 1:1000)
Phospho-EGF Receptor (Tyr1068) (D7A5) XP® Rabbit mAb (3777s, Cell Signaling Technology, 1:1000)
PD-L1 (E1L3N®) XP® Rabbit mAb (13684s, Cell Signaling Technology, 1:1000)
Recombinant Anti-CD47 antibody (ab218810, Abcam, 1:1000)
PD-L1 (D4H1Z) Rabbit mAb (60475s, Cell Signaling Technology, 1:1000)
Peroxidase AffiniPure Goat Anti-Rat IgG (H+L) (112-035-167, Jackson ImmunoResearch, 1:5000)
PD-L1 (D5V3B) Rabbit mAb (64988s, Cell Signaling Technology, 1:1000)
PD-L1/CD274 Polyclonal antibody (17952-1-AP, Proteintech, 1:100)
CD8α (D4W2Z) XP® Rabbit mAb (98941s, Cell Signaling Technology, 1:100)
FOLR1 Polyclonal antibody (23355-1-AP, Proteintech, 1:1000)
FR Antibody (E-11) (sc-515521, Santa Cruz Biotechnology, 1:500)
FOLR2 Polyclonal antibody (PA5-45768, Invitrogen, 1:500)
Goat anti-Mouse IgG (H+L) Alexa Fluor 488 (A11001, Invitrogen, 1:50)
Goat anti-Rabbit IgG (H+L) Cross-Adsorbed Secondary Antibody, Alexa Fluor™ 594 (A-11012, Invitrogen, 1:50)
Anti-rabbit IgG, HRP-linked Antibody (7074s, Cell Signaling Technology, 1:5000)
Anti-mouse IgG, HRP-linked Antibody (7076s, Cell Signaling Technology, 1:5000)

Validation

All antibodies were validated according to the manufacturer’s website and by assays done in this study.

Eukaryotic cell lines

Policy information about cell lines and Sex and Gender in Research

Cell line source(s)

B16F10, HepG2, Huh7, Hela, MDA-MB-231 and A549 were obtained from the American Tissue Culture Collection (ATCC). Fadu, SW480, Cal27, HACAT, CT26, MOC1, TU138 were obtained from UW-Madison Head Neck Cancer SPORE Pathology Core.

Authentication

Cell lines were authenticated by the suppliers.

Mycoplasma contamination

Cell lines were not tested for mycoplasma contamination.

Commonly misidentified lines (See ICLAC register)	No commonly misidentified cell lines were used in the study.

Palaeontology and Archaeology

Specimen provenance	*Provide provenance information for specimens and describe permits that were obtained for the work (including the name of the issuing authority, the date of issue, and any identifying information). Permits should encompass collection and, where applicable, export.*
Specimen deposition	*Indicate where the specimens have been deposited to permit free access by other researchers.*
Dating methods	*If new dates are provided, describe how they were obtained (e.g. collection, storage, sample pretreatment and measurement), where they were obtained (i.e. lab name), the calibration program and the protocol for quality assurance OR state that no new dates are provided.*

☐ Tick this box to confirm that the raw and calibrated dates are available in the paper or in Supplementary Information.

Ethics oversight	*Identify the organization(s) that approved or provided guidance on the study protocol, OR state that no ethical approval or guidance was required and explain why not.*

Note that full information on the approval of the study protocol must also be provided in the manuscript.

Animals and other research organisms

Policy information about studies involving animals; ARRIVE guidelines recommended for reporting animal research, and Sex and Gender in Research

Laboratory animals	BALB/c and C57BL/6 mice, female, 6-8 weeks old. BALB/c and C57BL/6 mice were used for tumor growth experiments. BALB/c mice were used for in vivo mechanism study. C57BL/6 mice were used for PK study. . Mice were group-housed, with no more than five mice per cage. Environmental conditions were maintained at a temperature of 22-25°C and a humidity level of 40-60%. The mice were kept on a 12-hour light/dark cycle with ad libitum access to food and water.
Wild animals	No wild animals were involved in this study.
Reporting on sex	female; Sex was not considered in this study as the goal was to demonstrate the application's technical capabilities.
Field-collected samples	This study did not involve field-collected samples.
Ethics oversight	All animal work experiments were approved by the University of Wisconsin-Madison Institutional Animal Care and Use Committee (IACUC) and conducted in accordance with the NIH Guide for the care and use of laboratory animals (animal protocol number M006396).

Note that full information on the approval of the study protocol must also be provided in the manuscript.

Clinical data

Policy information about clinical studies

All manuscripts should comply with the ICMJEguidelines for publication of clinical research and a completedCONSORT checklist must be included with all submissions.

Clinical trial registration	*Provide the trial registration number from ClinicalTrials.gov or an equivalent agency.*
Study protocol	*Note where the full trial protocol can be accessed OR if not available, explain why.*
Data collection	*Describe the settings and locales of data collection, noting the time periods of recruitment and data collection.*
Outcomes	*Describe how you pre-defined primary and secondary outcome measures and how you assessed these measures.*

Dual use research of concern

Policy information about dual use research of concern

Hazards

Could the accidental, deliberate or reckless misuse of agents or technologies generated in the work, or the application of information presented in the manuscript, pose a threat to:

No	Yes	
☐	☐	Public health
☐	☐	National security
☐	☐	Crops and/or livestock
☐	☐	Ecosystems
☐	☐	Any other significant area

Experiments of concern

Does the work involve any of these experiments of concern:

No	Yes	
☐	☐	Demonstrate how to render a vaccine ineffective
☐	☐	Confer resistance to therapeutically useful antibiotics or antiviral agents
☐	☐	Enhance the virulence of a pathogen or render a nonpathogen virulent
☐	☐	Increase transmissibility of a pathogen
☐	☐	Alter the host range of a pathogen
☐	☐	Enable evasion of diagnostic/detection modalities
☐	☐	Enable the weaponization of a biological agent or toxin
☐	☐	Any other potentially harmful combination of experiments and agents

Plants

Seed stocks	*Report on the source of all seed stocks or other plant material used. If applicable, state the seed stock centre and catalogue number. If plant specimens were collected from the field, describe the collection location, date and sampling procedures.*
Novel plant genotypes	*Describe the methods by which all novel plant genotypes were produced. This includes those generated by transgenic approaches, gene editing, chemical/radiation-based mutagenesis and hybridization. For transgenic lines, describe the transformation method, the number of independent lines analyzed and the generation upon which experiments were performed. For gene-edited lines, describe the editor used, the endogenous sequence targeted for editing, the targeting guide RNA sequence (if applicable) and how the editor was applied.*
Authentication	*Describe any authentication procedures for each seed stock used or novel genotype generated. Describe any experiments used to assess the effect of a mutation and, where applicable, how potential secondary effects (e.g. second site T-DNA insertions, mosiacism, off-target gene editing) were examined.*

ChIP-seq

Data deposition

☐ Confirm that both raw and final processed data have been deposited in a public database such as GEO.

☐ Confirm that you have deposited or provided access to graph files (e.g. BED files) for the called peaks.

Data access links<br>*May remain private before publication.*	*For "Initial submission" or "Revised version" documents, provide reviewer access links. For your "Final submission" document, provide a link to the deposited data.*
Files in database submission	*Provide a list of all files available in the database submission.*
Genome browser session<br>(e.g. UCSC)	*Provide a link to an anonymized genome browser session for "Initial submission" and "Revised version" documents only, to enable peer review. Write "no longer applicable" for "Final submission" documents.*

Methodology

Replicates	*Describe the experimental replicates, specifying number, type and replicate agreement.*
Sequencing depth	*Describe the sequencing depth for each experiment, providing the total number of reads, uniquely mapped reads, length of reads and whether they were paired- or single-end.*
Antibodies	*Describe the antibodies used for the ChIP-seq experiments; as applicable, provide supplier name, catalog number, clone name, and lot number.*
Peak calling parameters	*Specify the command line program and parameters used for read mapping and peak calling, including the ChIP, control and index files used.*

Data quality	*Describe the methods used to ensure data quality in full detail, including how many peaks are at FDR 5% and above 5-fold enrichment.*
Software	*Describe the software used to collect and analyze the ChIP-seq data. For custom code that has been deposited into a community repository, provide accession details.*

Flow Cytometry

Plots

Confirm that:

- [x] The axis labels state the marker and fluorochrome used (e.g. CD4-FITC).
- [x] The axis scales are clearly visible. Include numbers along axes only for bottom left plot of group (a 'group' is an analysis of identical markers).
- [ ] All plots are contour plots with outliers or pseudocolor plots.
- [x] A numerical value for number of cells or percentage (with statistics) is provided.

Methodology

Sample preparation	The expression of cell surface FR was analyzed by flow cytometry. Briefly, flow buffer was prepared as 2% FBS in PBS. 400,000 cells were harvested, washed with PBS, and then re-suspended in 400 µL ice-cold flow buffer containing 10 µM folate-FITC to incubate for 1 h at 37 °C. Cells were than washed twice with 1 mL ice-cold flow buffer and resuspended in 500 µL flow buffer with 1 µg/mL DAPI before flow analysis.
Instrument	ThermoFisher Attune NxT
Software	ThermoFisher Attune NxT cytometric software (v 6). FlowJo (v 10.8.1) was used for flow cytometry data analysis.
Cell population abundance	N/A
Gating strategy	Gating was based on FSC and SSC area, followed by selecting singlet using SSC-H vs SSC-A. Live cells were selected based on DAPI staining.

- [x] Tick this box to confirm that a figure exemplifying the gating strategy is provided in the Supplementary Information.

Magnetic resonance imaging

Experimental design

Design type	*Indicate task or resting state; event-related or block design.*
Design specifications	*Specify the number of blocks, trials or experimental units per session and/or subject, and specify the length of each trial or block (if trials are blocked) and interval between trials.*
Behavioral performance measures	*State number and/or type of variables recorded (e.g. correct button press, response time) and what statistics were used to establish that the subjects were performing the task as expected (e.g. mean, range, and/or standard deviation across subjects).*

Acquisition

Imaging type(s)	*Specify: functional, structural, diffusion, perfusion.*
Field strength	*Specify in Tesla*
Sequence & imaging parameters	*Specify the pulse sequence type (gradient echo, spin echo, etc.), imaging type (EPI, spiral, etc.), field of view, matrix size, slice thickness, orientation and TE/TR/flip angle.*
Area of acquisition	*State whether a whole brain scan was used OR define the area of acquisition, describing how the region was determined.*

Diffusion MRI ☐ Used ☐ Not used

Preprocessing

Preprocessing software	*Provide detail on software version and revision number and on specific parameters (model/functions, brain extraction, segmentation, smoothing kernel size, etc.).*
Normalization	*If data were normalized/standardized, describe the approach(es): specify linear or non-linear and define image types used for transformation OR indicate that data were not normalized and explain rationale for lack of normalization.*

Normalization template

Describe the template used for normalization/transformation, specifying subject space or group standardized space (e.g. original Talairach, MNI305, ICBM152) OR indicate that the data were not normalized.

Noise and artifact removal

Describe your procedure(s) for artifact and structured noise removal, specifying motion parameters, tissue signals and physiological signals (heart rate, respiration).

Volume censoring

Define your software and/or method and criteria for volume censoring, and state the extent of such censoring.

Statistical modeling & inference

Model type and settings

Specify type (mass univariate, multivariate, RSA, predictive, etc.) and describe essential details of the model at the first and second levels (e.g. fixed, random or mixed effects; drift or auto-correlation).

Effect(s) tested

Define precise effect in terms of the task or stimulus conditions instead of psychological concepts and indicate whether ANOVA or factorial designs were used.

Specify type of analysis: ☐ Whole brain ☐ ROI-based ☐ Both

Statistic type for inference

(See Eklund et al. 2016)

Specify voxel-wise or cluster-wise and report all relevant parameters for cluster-wise methods.

Correction

Describe the type of correction and how it is obtained for multiple comparisons (e.g. FWE, FDR, permutation or Monte Carlo).

Models & analysis

n/a	Involved in the study
☐	☐ Functional and/or effective connectivity
☐	☐ Graph analysis
☐	☐ Multivariate modeling or predictive analysis

Functional and/or effective connectivity

Report the measures of dependence used and the model details (e.g. Pearson correlation, partial correlation, mutual information).

Graph analysis

Report the dependent variable and connectivity measure, specifying weighted graph or binarized graph, subject- or group-level, and the global and/or node summaries used (e.g. clustering coefficient, efficiency, etc.).

Multivariate modeling and predictive analysis

Specify independent variables, features extraction and dimension reduction, model, training and evaluation metrics.
